# Impact of urbanization on exposure to extreme warming in megacities

**DOI:** 10.1016/j.heliyon.2023.e15511

**Published:** 2023-04-18

**Authors:** Do Ngoc Khanh, Alvin C.G. Varquez, Manabu Kanda

**Affiliations:** Department of Transdisciplinary Science and Engineering, Tokyo Institute of Technology, 2-12-1 Ookayama, Meguro, Tokyo, 152-8550, Japan

**Keywords:** Global warming, Urbanization, Urban heat island, Megacity, Exposure

## Abstract

Cities warm up due to two main factors: global climate change and urbanization-induced warming (so-called, urban heat island effect). In the projection of future climate, coarse-resolution global climate models are not suitable for looking into the heterogeneous urban surface and their changes. On the other hand, regional climate models, which are capable of looking into cities in detail, have never been used to investigate the global urban climate. Here we show that urbanization significantly increases exposure to extreme warming for megacity residents. We reflect urbanization between the 2010s and the 2050s into our model by considering the spatiotemporal change in urban surface (buildings and anthropogenic heat emissions) induced by urban population and economic growth. We found that in the 2050s, under the worst-case scenario, 78 percent of megacity residents will be exposed to 2.5 °Cwarming, much higher than the projection of 65 percent when urban warming is left out. Our results highlight the importance of accounting for local urbanization in future global urban climate projection.

## 1 Introduction

Cities around the world are forming and expanding rapidly. It is projected that 60 percent of world population will settle in urban areas by 2030. Moreover, just 43 megacities will house 8.8 percent of world population [1]. Simultaneously, global warming of 1.5 °Cis likely to be reached between 2030 and 2052 [2]. Change in land cover, buildings, and heat released from human activities are major causes of urban heat island—the phenomenon that urban areas are warmer than its rural surrounding [3]. As global warming progresses and cities continue to growth both horizontally and vertically, understanding the interaction and combined effects of global warming and urbanization on urban climate and on urban settlers are crucial for planning resilient cities and ensure a sustainable future.

Climatological study for cities has been receiving more and more attention in recent years because of the great concentration of population and the contribution of urban heat island to city warming [4], [5], [6]. Urban climatology is a tool to understand the past, present, and future urban climate itself [7], [8], [9], [10], [11], [12], [13] and to project heat-related risks to human [14], [15], [16]. Future climate projection at a global scale can be done by running general circulation models (GCMs) under various emission scenarios. However, GCMs, even with bulk parameterization of urban surface [17], are not satisfactory for climatological study at city scale due to their coarse resolution. Instead, regional climate models (RCM) coupled with urban canopy schemes [18], [19], [20] are suitable for study at city scale. They have been used to investigate urban heat island [21], [22], precipitation [23], [24], and found potential in operational weather forecasting [25]. In addition, using RCM to downscale GCMs' projection is one method to project future city climate.

Projection for cities around the world has been conducted but there are some remaining issues, specifically on the following three points:

1. There are three important factors in urban modeling which are the present and futuristic spatially distributed projection of (1) urban morphological parameters (e.g., building height, plan area index, and roughness length), (2) urban expansion, and (3) anthropogenic heat emission. However, in past studies, these parameters were only partially considered. For example, one global study [17] considered anthropogenic heat but not heterogeneous urban morphological parameters nor urban expansion; a study for Tokyo [7] considered spatially varying anthropogenic emission but used constant urban morphological parameters; a study for Sydney [8] considered urban expansion but completely ignored anthropogenic heat; a study for Hanoi [11] considered urban expansion and anthropogenic heat but did not mention urban morphological parameters (e.g., building height and roughness length). That is to say, the three factors have been considered more and more thoroughly, but they have not been considered fully yet.
2. It is not straightforward to generalize the methodologies of past studies due to their dependence on locally available datasets. For example, the study for Sydney [8] used local land use dataset, the study for Hanoi [11] used city future master plan, and the study for Tokyo [7] used national energy use dataset. These local datasets are good for the concerned cities but they are not suitable for a multi-city study because they have not been available publicly for many cities worldwide yet. Moreover, it is difficult to collect and unite datasets of different format and not all cities have data and future plan published for a specific year or period (e.g., the 2050s).
3. In relation to the issues 1 and 2, there is additionally a geographical bias in urbanization and climate change study according to a review [26]. Specifically, while many studies were conducted for European, North American, and Chinese cities, few were conducted for the vulnerable South American and African cities. In addition to the geographical bias, the review also pointed out that only 14% of reviewed articles examined the combined effect of climate change and urban heat island and only 16% examined consequences to urban population. Recently, more studies [27], [28] have been done to address the geographical bias; however, the aforementioned issues (e.g., lack of consideration of urbanization and/or anthropogenic heat) persist.

To address all the three issues mentioned above, we generalize a method previously used for Jakarta, a tropical megacity [10]. Our target is to project the future climate for all 43 megacities (Table 1) classified by the United Nations (including African and South American cities, addressing issue 3), using only public databases and open source software (addressing issue 2), with consideration of present and future spatially varying urban morphological parameters, urban expansion, and anthropogenic heat emission (addressing issue 1). We focus on two aspects of urban warming (global warming and urbanization-induced warming) and examine possible consequences to urban dwellers (addressing issue 3). We show that urbanization can drastically speed up the warming in cities and significantly increase the number of people facing extreme warming. Because all datasets are global, this approach can be adapted to any region of interest, not limited to megacities.

**Table 1:** List of 43 megacities [1]. The city code of each city is taken from the IATA metropolitan area code or the code of a major international airport in the city.

City	Code	City	Code	City	Code	City	Code
Ahmadabad	AMD	Delhi	DEL	Lahore	LHE	Osaka	OSA
Bangalore	BLR	Dhaka	DAC	Lima	LIM	Paris	PAR
Bangkok	BKK	Guangzhou	CAN	London	LON	Rio de Janeiro	RIO
Beijing	PEK	Ho Chi Minh City	SGN	Los Angeles	LAX	Seoul	SEL
Bogota	BOG	Hyderabad	HYD	Luanda	LAD	Shanghai	SHA
Buenos Aires	BUE	Istanbul	IST	Manila	MNL	Shenzhen	SZX
Cairo	CAI	Jakarta	JKT	Mexico City	MEX	São Paulo	SAO
Chengdu	CTU	Karachi	KHI	Moscow	MOW	Tehran	THR
Chennai	MAA	Kinshasa	FIH	Mumbai	BOM	Tianjin	TSN
Chongqing	CKG	Kolkata	CCU	Nanjing	NKG	Tokyo	TYO
Dar es Salaam	DAR	Lagos	LOS	New York	NYC		

## 2 Methods

We study the worst-case scenario by coupling the Shared Socioeconomic Pathways 3 (SSP3) and the Representative Concentration Pathway 8.5 (RCP8.5). Hereinafter, for conciseness, we use 2010 and 2050 to refer to the 2006–2015 and the 2046–2055 decade, respectively. Our projection consists of two steps: estimating the warming between the pre-industrial era and 2010 and projecting the warming between 2010 and 2050. The final projection is the summation of the two steps.

We estimate the warming between the pre-industrial era (1850–1900) and 2010 (i.e., 2006–2015 decade) by ensemble averaging and time averaging the near-surface air temperature variable (tas) of five GCM members (GFDL-ESM2M, IPSL-CM5A-LR, MIROC-ESM-CHEM, HadGEM2-ES, and NorESM1-M) of the Coupled Model Intercomparison Project 5 (CMIP5) for each period and then take the difference between the two. We used the data from the historical run for the pre-industrial period and from the RCP8.5 run for the 2006–2015 period. The estimated warming level for each city is shown in Table A.2. We note that the choice of CMIP5 instead of the newer CMIP6 is to maintain consistency with the assumptions of other datasets [29], [30] used in this study.

Below we describe our methods for projecting the warming between 2010 and 2050. We used a modified version of the WRF model v3.3.1 coupled with a single-layer urban canopy model [31] which has been validated in previous studies [32], [10]. Compared to the original single-layer urban canopy model [19] which handles three urban categories (low density residential, high density residential, and commercial), in this modified version, each urban grid is modeled by five morphological parameters: average building height $\left(H_{avg}\right)$, plan area index $\left(\lambda_{p}\right)$, frontal area index $\left(\lambda_{f}\right)$, roughness length $\left(z_{0}\right)$, and zero-plane displacement height (*d*). In addition, hourly spatially varying anthropogenic heat is emitted to the first atmospheric level and is not a part of the urban canopy model calculation. Detailed model setup is given in Table 2. The model code is available in GitHub (https://github.com/TokyoTechGUC/WRF-distributed-urban).

**Table 2:** Model setup.

Domains	One-way nesting with a fine domain (2 km resolution) nested in a coarse domain (10 km resolution)
Sea surface temperature update	Disable
Adaptive time step	Enable
Microphysics	New Thompson et al. scheme
Longwave radiation	Rapid Radiative Transfer Model (RRTM) scheme
Shortwave radiation	Goddard shortwave
Surface layer	MM5 similarity
Land surface	Noah land surface model
Urban surface	Single layer urban canopy model with distributed urban morphological parameters
Planetary boundary layer	Mellor-Yamada Nakanishi and Nitto Level 2.5
Cumulus parameterization	Kain-Fritsch scheme for the coarse domain

### 2.1 Urban surface modeling

Country-level gross domestic product (GDP) and population projection under SSP3 needed as input for projecting future population density and urban morphological parameters were downloaded from the SSP Public Database [33], [34], [35], [36]. The population density in the present was calculated from the LandScan 2013 dataset [37]. The population density in the future was projected by combining a logistic model with a global urban sprawling map [30] and applying adjustment by nighttime lights. The method for projecting future population density was explained in detail in a previous publication [29].

Next, using the population map, we modified the 20-class 500 m MODIS land cover dataset included in the WRF model. For the present simulation, to put more emphasis on urban land use, all MODIS grids with the population count of at least 1000 are assigned to urban category. For the future simulation, all MODIS grids with the population density of at least 1000 per sq. km are assigned to urban category.

To parameterize the urban surface, from GDP and population density information, we used empirical formulas to estimate $H_{avg}$, $\lambda_{p}$ [38], and then $\lambda_{f}$, $z_{0}$, and *d* [39]. The formulas can be found in the corresponding references or Appendix A.2. The resolution of the urban morphological parameters map is 1 km by 1 km. We also used the present and future 1 km hourly anthropogenic heat flux AH4GUC dataset [29]. Calculated urban morphological parameters are available on figshare [40] and summarized in Table A.2.

### 2.2 Reproducing the present climate condition

To know the average weather condition in a city in a certain month through out the present period (2006–2015), a conventional method is to run a 10-year simulation and then ensemble average the output data by month. However, this method (average after simulation) is computationally expensive, thus, we attempted to use a cheaper method (simulation after average).

We explain in detail the process of obtaining the average weather condition for January over 2016–2015 period. The process for other months of the year is similar. First, we downloaded the National Centers for Environmental Prediction (NCEP) Final Operational Global Analysis (FNL) dataset for all hours and all days in January in the 2006–2015 period. Next, for each available hour of the day (00, 06, 12, and 18 UTC), we ensemble averaged all the FNL files of that hour in all Januaries of the decade. This operation resulted in a set of four meteorological input files which is enough for a one-day simulation. We regard this one day as a typical January day of the decade. We then assumed that this typical day repeats for four consecutive days and made four copies of the four meteorological input files so that we could run a four-day simulation. The first day of simulation was for model spin-up and its output was discarded. The remaining three-day simulation output was ensemble averaged to a one-day output which was used for further analysis. This simulation method is summarized graphically in Fig. A.2a was also applied in previous test runs [41], [42].

### 2.3 Projecting the future climate condition

Meteorological input for simulation of the future was constructed using the pseudo-global warming (PGW) method [43]. In this method, future meteorological input is assumed to be the sum of the present meteorological input and the difference between GCMs' outputs of the present and the future. In this study, we downloaded the output of the five GCM members of CMIP5 mentioned above for the 2006–2015 decade and the 2046–2055 decade (RCP8.5 run). The difference between the ensemble average of the two decades was added to the present meteorological input to generate the future meteorological input. Specifically, we added the difference in wind velocity components, surface temperature (which includes sea surface temperature), air temperature, and geopotential height (Fig A.2b). Using the generated future meteorological input, simulations for each city and each month were conducted similar to simulations for the present.

### 2.4 Simulation scenarios

With the urban surface modeling and simulation procedures described above, we constructed three simulation scenarios (Table 3). The present scenario is used as hindcasting to evaluate model performance. The difference between the present and the intermediate scenario is used to assess the impact of global warming and the difference between the intermediate scenario and the future scenario is used to assess the impact of urbanization. We conducted one simulation for each city, each month of the year, and each scenario on the TSUBAME supercomputer.

**Table 3:** Simulation scenarios.

Scenario	Urban surface	Meteorological input
Present	present	present
Intermediate	present	future
Future	future	future

## 3 Results and discussion

### 3.1 Model verification

The hindcasting result was verified against observation data downloaded from the NOAA Integrated Surface Database (ISD) and shown in Table A.1. To save computational resources, we inputted the ensemble average of historical weather data to the model, instead of using the common approach of inputting historical weather data to the model and then ensemble averaging simulation outputs. However, we obtained a good correlation between the simulated temperature and the ensemble average of observed temperature. Additionally, model bias has little effect because we only consider the difference between simulations. Thus, we conclude that the model performance is adequate for further discussion.

### 3.2 Urbanization-induced warming

For conciseness, we use the term “urbanization” to refer to projected urbanization between 2010 and 2050, and use the term “warming level” to refer to the increase in air temperature from the pre-industrial period. We project the spatial distribution of warming level in 2050 under two scenarios: with and without urbanization. Here, urbanization covers urban expansion, population change, and economic growth which in turn induces changes in urban morphology and anthropogenic heat emission in the model. The change in heat emission can be negative due to population decline.

**Figure 1:**
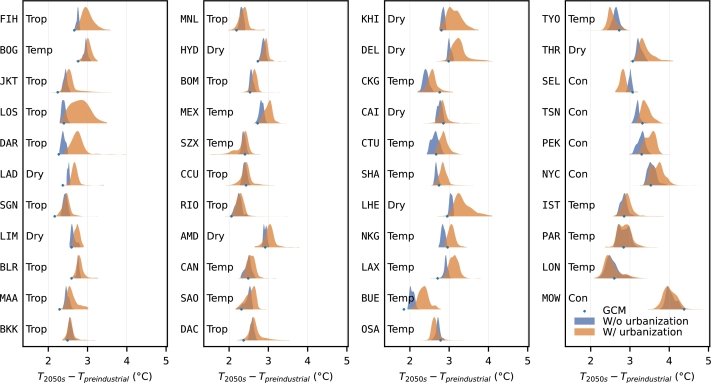
Kernel density estimate of 2 km by 2 km gridded warming level in 2050 (*T*_2050*s*_ − *T*_*preindustrial*_) when urbanization between 2010 and 2050 is not considered (blue shaded area) and when it is considered (orange shaded area) in each of the 43 megacities. The average projection of five GCM members of CMIP5 is indicated by blue dots. Refer to Table 1 for city codes. Cities are sorted in the ascending order of latitude (from top to bottom, then, from left to right). Trop, Dry, Temp, and Con represent the Köppen climate classification of tropical, dry, temperate, and continental, respectively [44]. See also Fig. A.1 for a detailed graphical explanation.

The spatial distributions of the warming level in the 43 megacities are plotted using kernel density estimators together with the mean projection of five GCM members of CMIP5 in Fig. 1. In each city, the projection of the GCM members of CMIP5 can either fall on the left, on the right, or in the two distributions. That is to say GCM projection of warming level may be either higher or lower than downscaled RCM projection of warming level at urban areas. This difference suggests that using only GCM to project warming level of cities may be insufficient. Without considering the effect of urbanization, the spatial variance of warming level in the megacities is relatively small as indicated by the narrow widths of the blue shaded areas in Fig. 1. However, when urbanization is taken into account, the spatial distribution of warming level changes drastically. Together with the growth of the cities, building coverage, building height, and energy usage increases. These factors strengthen the urban heat island effect, making cities warm up faster than the regional and global average. Moreover, because cities do not develop uniformly, the speed of warming varies from location to location. In Fig. 1, the warming effect induced by urbanization is indicated by the shift of the warming level distribution from the blue shaded area to the orange shaded area. For example, using the rate of change of urban morphological parameters and anthropogenic heat emission (Table A.2) as a proxy for the speed of urbanization, shifts to the right can be seen clearly in fast growing cities such as Kinshasa, Lima, and Chengdu. On the contrary, for highly developed cities that will be under population decline such as Tokyo, Osaka, and Seoul, incorporating projection of energy usage change results in milder warming than the warming projection without considering energy usage change (i.e., shifts to the left). Also in Fig. 1, the non-uniformity of the development speed within each city is reflected in the large variance of the warming level distribution. The warming level distribution of Lahore is given in Fig A.1b as an example.

**Figure 2:**
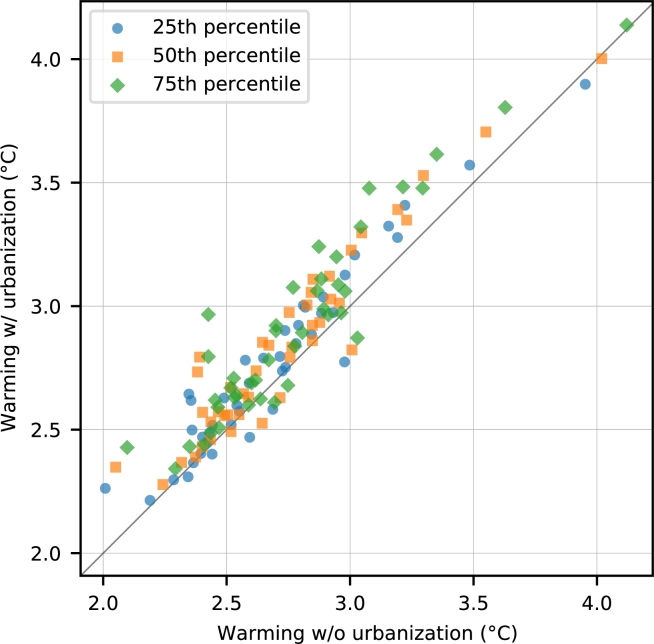
The first, second, and third quartiles (25th, 50th, and 75th percentiles) of the gridded warming level in 2050 when urbanization is not considered (horizontal axis) and when it is considered (vertical axis) in each of the 43 megacities. Thus, each city has three dots comparing the three quartiles of the corresponding blue and orange distribution in Fig. 1.

Quantitatively, the average warming due to urbanization of each city varies from -0.17 to 0.40 °Cwith the mean and standard deviation of 0.12 and 0.12 °C, respectively. This large variance reflects the difference in urbanization speed among the cities. Additionally, the interquartile range of the gridded warming projection incorporating the urbanization effect of each city varies from 0.08 to 0.35 °Cwith the mean value of 0.15 °C. These values demonstrate the difference in warming speed even within the border of a single city. Comparing the quartiles of the gridded warming projection (Fig. 2), we found that in general, the projection without consideration of urbanization is lower than the projection with consideration of urbanization in all quartiles and the deviation grows as we move from the lower quartile to the upper quartile. Specifically, the average deviation for the first, second, and third quartiles are 0.08, 0.11, and 0.14 °C, respectively (see also Fig. 2). Thus, without considering urbanization, heat-related risks maybe underestimated, especially when the focus is on the upper quartile.

### 3.3 Warming and megacity dwellers

The number of megacity dwellers affected by a certain warming level is controlled by three factors: present population, population growth, and urban warming. Urbanization is related to the last two factors. In this section, we attempt to quantify the contribution of each of the three factors. For concreteness and simplicity, we use 2.5 °Cwarming as an example to describe the calculation method. First, we overlaid the present population map with the projected warming level without urbanization map and counted the total number of people exposed to 2.5 °Cwarming (i.e., the number of people living at grids that have 2.5 °Cincrease in temperature). This is the number of people exposed to the warming assuming no population, urban, or energy usage growth (first row of Table 5a). Thus, we regard it as the contribution of present population. Next, we overlaid the future population map with the projected warming level without urbanization map and repeated the count. This is the number of people exposed to the warming assuming population growth but no urban or energy usage growth (thus, no urban warming; second row of Table 5a). The difference between this number and the contribution of the present population is the contribution of population growth. Finally, we did the count for the future population map overlaid on the projected warming level with urbanization map. This is the number of people exposed to the warming when population growth and urban warming are both accounted (third row of Table 5a). Subtracting the contribution of present population and population growth from this number, we obtained the contribution of urban warming. As shown in Table 5b, 612 million megacity dwellers are projected to be exposed to 2.5 °Cwarming with the total figure broken down to three contributors as: present population (427 mil., 70%), population growth (81 mil., 13%), and urban warming (104 mil., 17%).

**Figure 3:**
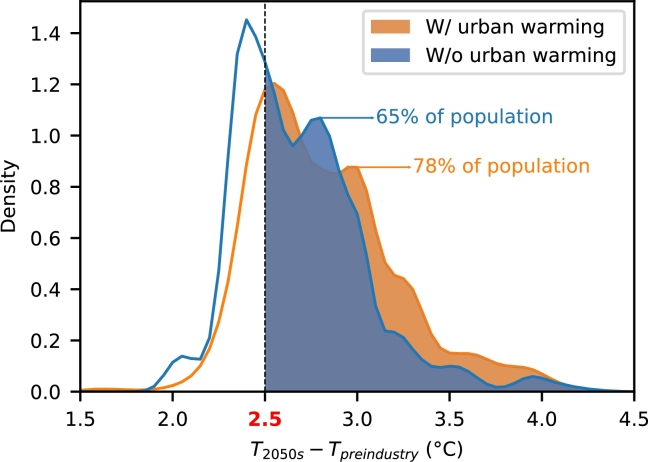
Warming level in 2050 (with and without consideration of urban warming) plotted against the number of people who will experience it. The plot is smoothed by a Gaussian kernel density estimator. The area under each curve represents 782 million people from 43 megacities in 2050.

Next, we focus on the impact of the urban warming factor. The projection of the number of people exposed to different warming levels when urban warming is accounted/unaccounted for is shown in Fig. 3 and Table 4. Regardless of the consideration of urban warming, almost 100% of the population in megacities will be exposed to 2.0 °Cwarming. Without considering urban warming, 65% of the megacity population will be exposed to 2.5 °Cwarming. By considering urban warming, another 13% will be exposed. Focusing on extreme warming of 3.0 and 3.5 °C, we found that the number of affected people is almost doubled when urban warming is taken into account. Moreover, as shown in Table 5b, the contribution of the urban warming factor grows together with the extremeness of the warming level, from 0% in 2.0 °Cwarming to 17% in 2.5 °Cwarming and 49% in 3.5 °Cwarming. Therefore, limiting urban warming (e.g., finding optimal city layout, reducing anthropogenic heat) has a potential to reduce the number of people affected by extreme warming. However, finding a mitigation is not in the scope of this study. Note that all the numbers discussed so far are summations across all the megacities.

**Table 4:** Percentage of population in megacities affected by various warming levels. The base is 782 million people from 43 megacities in 2050.

Projection	Warming level			
	≥ 2.0 °C	≥ 2.5 °C	≥ 3.0 °C	≥ 3.5 °C
Without urban warming	99%	65%	15%	3%
With urban warming	100%	78%	29%	7%

**Table 5:** **a** The number of people (million) in megacities exposed to various warming levels under different considerations of urbanization. + indicates an included factor. **b** Contribution of each factor (million of people and percentage) into the exposure to various warming levels.

**a**	
	**Table:** Factors Warming level Present pop. Pop. growth Urban warming ≥ 2.0 °C ≥ 2.5 °C ≥ 3.0 °C ≥ 3.5 °C + 606 427 108 26 + + 778 508 121 27 + + + 778 612 226 53

**b**	
	**Table:** Factor Warming level ≥ 2.0 °C ≥ 2.5 °C ≥ 3.0 °C ≥ 3.5 °C Present pop. 606 (78%) 427 (70%) 108 (48%) 26 (49%) Pop. growth 172 (22%) 81 (13%) 13 (6%) 1 (2%) Urban warming 0 (0%) 104 (17%) 105 (46%) 26 (49%) Total 778 (100%) 612 (100%) 226 (100%) 53 (100%)

Risk of climate change to human has three components: hazard (a weather or climate event that can adversely affect people), vulnerability (level of preparedness to the hazard), and exposure (the presence of people in places that could be affected) [45], [46]. For example, the risk of heat stroke for megacity dwellers has three components: hazard (high air temperature), vulnerability (varying with socioeconomic factors such as population pyramid and availability of cooling system), and exposure (the number of people living at places with high air temperature). Our result indicates that urban warming increases the number of people living at places with high air temperature, thus, increases the exposure component. This may increase the risk of heat stroke in the future. However, the increase in risk is not directly proportional to the increase in exposure because risk also depends on the variation of the hazard component (for example, change in heat wave frequency [47]) and the vulnerability component (for example, vulnerability is increased due to an aging population but can be lowered by preventive measures [48]). The same argument applies for other kind of heat-related risks; therefore, we conclude that without considering urban warming, there is a possibility of underestimating heat-related risks for cities. A comprehensive study accounting for many climatological and socioeconomical factors may be done in the future to fully project future heat-related illness risks.

## 4 Concluding remarks

In conclusion, we projected the future climate of 43 megacities in the 2046–2055 decade under the worst-case scenario of RCP8.5 and SSP3 with consideration of changing urban morphological parameters and anthropogenic heat emission due to urbanization. The major findings are

• The effect of urbanization has small mean compared to the effect of global climate change. However, urbanization effect varies significantly from location to location even within the border of one city. Therefore, the spatial variance of the urbanization effect is possibly more important than the mean.
• 78% of megacity population (612 million people) may be exposed to 2.5 °Cwarming, in which 17% is due to urban warming induced by urbanization from the 2010s to the 2050s. Therefore, urbanization effect should be included in projections of future heat-related illness risks because the effect may increase the risks.

Limitations and possible future research directions to address those limitations are

• We only made projection for the worst-case scenario while projections for other scenarios are equally important. They can be done by adjusting the projections of urbanization and future energy usage inputted to the model.
• We did not conduct sensitivity analysis for the impact of urbanization. Even though uncertainty due to different urbanization pathways is significant smaller than the uncertainty due to emission scenarios or GCM projections in the case of a mature metropolis [49], the uncertainty for rapidly developing megacities in developing countries is unknown.
• Under the setting of this research, the term “impact of urbanization” should be read in full as “impact of urbanization projected under the future climate forcing of RCP8.5.” In reality, urbanization and global warming happens simultaneously, unlike the two-step process (first global warming and then urbanization) presented here.
• We utilized climate boundaries from CMIP5 datasets for consistency with urbanization scenarios and anthropogenic heat derived from past studies. However, given a generalized methodology presented herein, the findings may be advanced further by considering additional urbanization and climate-change scenarios, such as the increasingly available CMIP6 datasets.

Using our model and global datasets, further research can be done for many cities worldwide to

• investigate the effect of urbanization on meteorological variables other than temperature (for example, humidity, wind speed, and precipitation) and extreme weather events (for example, heat wave, cold wave, and typhoon),
• investigate the dependency of urban warming on climate forcing, climate zone, and atmospheric circulation regimes,
• analyze variations and similarities of urban warming among different cities,
• project various consequences of warming to human (for example, change in thermal comfort, heat-related risks, mosquito-borne diseases risks [50]).

We hope that our approach of using open source model and freely available global datasets will be especially useful for studying developing regions with little data available. We expect that along with global climatological research focusing on the whole globe and urban climatological research focusing on individual cities, more research examining city-scale phenomena from a global point of view will be conducted.

## CRediT authorship contribution statement

Do Ngoc Khanh: Conceived and designed the experiments; Performed the experiments; Analyzed and interpreted the data; Wrote the paper.

Alvin C. G. Varquez; Manabu Kanda: Conceived and designed the experiments; Analyzed and interpreted the data.

## Declaration of Competing Interest

The authors declare that they have no known competing financial interests or personal relationships that could have appeared to influence the work reported in this paper.

## Data Availability

Calculated urban morphological parameters are available on figshare (https://doi.org/10.6084/m9.figshare.17108981). Anthropogenic heat dataset (AH4GUC) and urban sprawl dataset are available from the corresponding references. The modified WRF model code is available on GitHub (https://github.com/TokyoTechGUC/WRF-distributed-urban). Other data are available from the corresponding author on reasonable request.

## Appendix A Supplementary material

MMC This supplementary file contains Appendix A. MMC
