# Supplementary material for: Impact of urbanization on exposure to extreme warming in megacities
Source: Heliyon. 2023 Apr 18;9(4):e15511. doi: 10.1016/j.heliyon.2023.e15511 (PMC10148033; doi:10.1016/j.heliyon.2023.e15511)
Supplement: MMC — This supplementary file contains Appendix A. [file mmc1.pdf]

A Supplementary information

A.1 Supplementary figures and tables

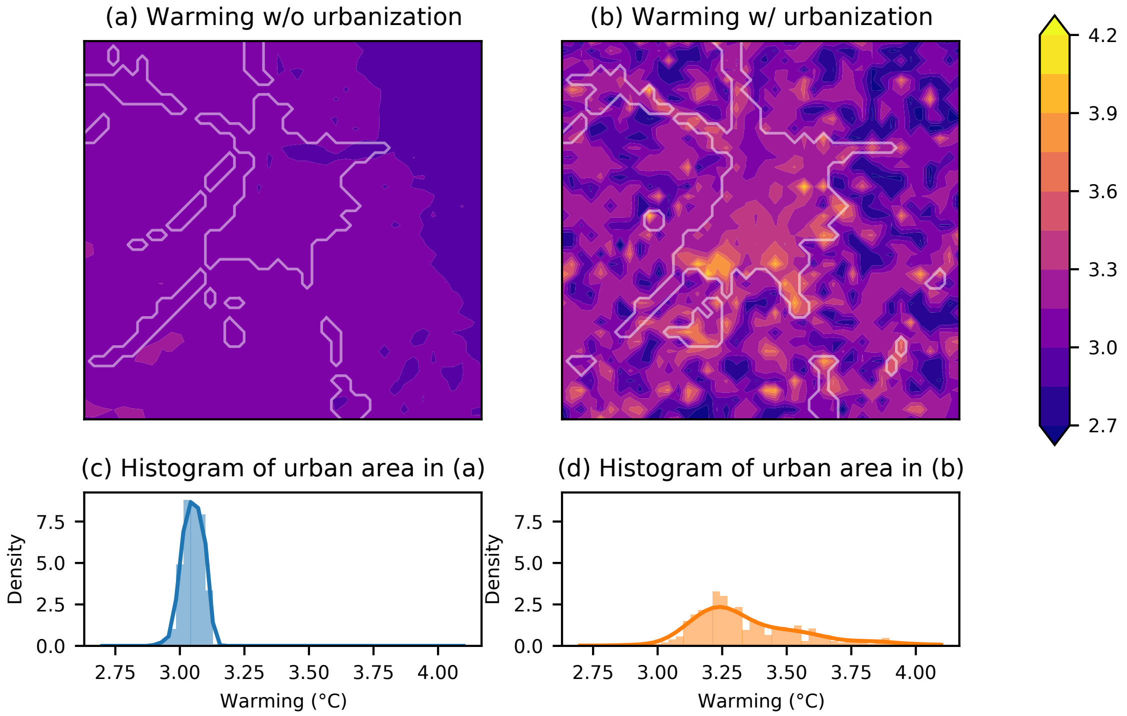

Figure A.1: Related to Fig. 1. Detailed explanation for Lahore (LHE). First, the spatial distribution of the warming without and with urbanization in consideration is plotted in (a) and (b). White lines depict the urban boundary in the present (a) and future (b). These boundaries are drawn based on the land use dataset and they are not administrative boundaries. Next, the normalized histogram of warming within the urban area and kernel density estimates are calculated and plotted in (c) and (d). The distributions shown in Fig. 1 are the areas below kernel density estimates (the blue and orange lines), the height of the two areas are adjusted to be the same for better visibility.

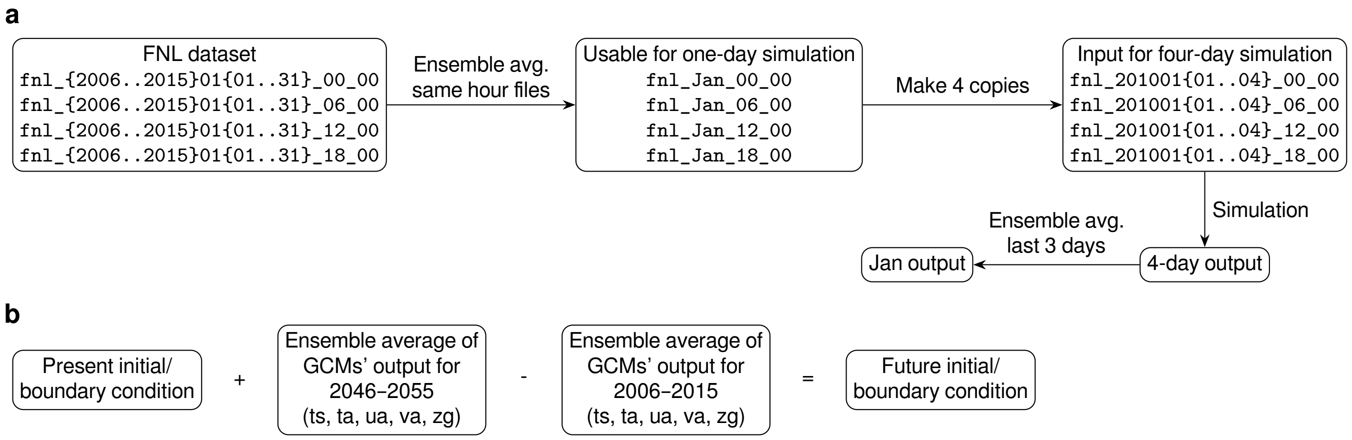

Figure A.2: **a** Simulation flow for January of the present scenario. Simulation for other months and other scenarios are conducted similarly. `{a..b}` is a shorthand for listing $a, a+1, \ldots, b$. **b** The method to prepare future meteorological input.

Table A.1: Verification of hindcasting simulation including the number of observations (n), root-mean-square error (RMSE), bias, and Pearson correlation coefficient (r). Each observation is the ensemble average of observed temperatures at one station, one time of the day, and one month throughout the 2006–2015 decade. Stations are grouped to urban and non-urban stations using the MODIS land use dataset, which is also inputted to the WRF model. Shenzhen (SHE) and São Paulo (SAO) are covered by Guangzhou (GUA) and Rio de Janeiro (RIO) domains, respectively. Refer to Table 1 for city codes. Station locations are shown in Appendix A.3.

	Urban stations				Non-urban stations			
City	n	RMSE	Bias	r	n	RMSE	Bias	r
AMD	96	1.54	0.90	0.98	0			
BKK	1176	1.26	0.43	0.93	672	0.97	-0.12	0.95
BLR	166	1.29	-0.49	0.95	0			
BOG	288	2.80	2.44	0.96	144	1.18	-0.41	0.87
BOM	192	1.63	0.88	0.96	0			
BUE	755	2.58	1.12	0.98	141	2.48	0.17	0.95
CAI	288	2.07	-0.28	0.98	0			
CAN	1632	2.36	1.81	0.96	384	1.95	1.43	0.97
CCU	220	1.99	0.77	0.96	27	1.85	-0.39	0.97
CKG	288	2.31	1.70	0.98	0			
CTU	288	2.29	2.02	0.99	96	2.49	2.05	0.98
DAC	215	2.47	1.36	0.95	0			
DAR	288	1.00	0.38	0.97	280	1.17	-0.56	0.95
DEL	384	2.26	1.17	0.98	0			
FIH	576	2.21	1.17	0.86	0			
HYD	96	1.27	0.81	0.98	0			
IST	228	2.23	-1.00	0.98	0			
JKT	840	1.38	0.83	0.96	0			
KHI	288	2.03	-0.17	0.96	0			
LAD	214	1.23	0.83	0.98	0			
LAX	144	2.71	-1.60	0.96	0			
LHE	384	2.86	1.42	0.99	126	2.76	0.94	0.98
LIM	288	1.76	1.51	0.96	0			
LON	1440	2.39	-0.52	0.98	864	2.01	-0.67	0.98
LOS	630	0.95	0.21	0.93	0			
MAA	96	1.43	0.12	0.95	0			
MEX	280	2.01	-0.98	0.93	84	1.41	0.39	0.96
MNL	1242	1.13	0.38	0.91	762	1.05	0.12	0.93
MOW	384	2.65	-1.03	0.99	576	3.09	-1.47	0.99
NKG	384	2.64	1.77	0.98	0			
NYC	408	2.37	0.17	0.98	568	2.17	0.05	0.98
OSA	3216	1.77	1.16	0.99	1308	1.57	0.95	0.99
PAR	1440	2.29	-0.11	0.99	1484	2.05	-0.39	0.99
PEK	288	2.50	0.97	0.99	0			
RIO	2892	1.98	0.83	0.91	685	1.50	0.64	0.95
SEL	1536	2.01	0.45	0.99	672	1.67	0.61	0.99
SGN	288	1.69	1.33	0.95	0			
SHA	672	1.95	1.14	0.99	384	1.34	-0.28	0.99
THR	480	2.09	-0.22	0.99	288	2.17	-0.06	0.99
TSN	264	2.59	1.48	0.99	288	2.51	0.84	0.99
TYO	5451	2.08	1.55	0.99	972	1.58	0.78	0.98

Table A.2: Average values of urban morphological parameters and anthropogenic heat emisison (AHE) in the 2010s and 2050s, and warming level in the 2010s ($\Delta T_{2010s} = T_{2010s} - T_{preindustrial}$) of the 43 megacities. The value in the 2050s are given in percentage increase from the value in the 2010s. Note that because present and future city boundaries are different, the spatial average values are taken within the present city boundary for a fair comparison. Refer to Table 1 for city codes.

City	H_{avg} (m)		λ_p		λ_f		z_0 (m)		d (m)		AHE (W/m^2)		ΔT_{2010s}
	2010	Δ2050	2010	Δ2050	2010	Δ2050	2010	Δ2050	2010	Δ2050	2010	Δ2050	(°C)
AMD	7.18	4.9%	0.11	3.1%	0.07	3.0%	0.49	10.8%	8.29	7.5%	3.56	108.1%	1.07
BKK	6.68	6.8%	0.11	6.4%	0.08	5.5%	0.56	13.1%	7.77	11.9%	5.14	3.1%	1.11
BLR	8.24	22.9%	0.13	1.9%	0.10	1.1%	0.96	41.6%	11.28	31.9%	4.66	126.7%	1.19
BOG	9.91	18.5%	0.17	14.6%	0.13	19.3%	1.16	41.6%	14.24	28.5%	9.25	49.0%	1.17
BOM	7.53	9.8%	0.11	0.3%	0.08	0.4%	0.59	21.1%	9.12	14.5%	5.14	109.3%	0.99
BUE	9.95	22.6%	0.16	12.9%	0.12	15.8%	1.03	45.0%	14.09	32.0%	10.44	77.9%	0.80
CAI	8.44	4.3%	0.14	2.8%	0.10	2.2%	0.77	7.0%	11.20	6.3%	8.78	23.6%	1.10
CAN	5.18	5.6%	0.08	0.3%	0.06	0.1%	0.30	15.4%	4.88	10.3%	3.76	120.5%	0.64
CCU	6.29	3.2%	0.09	1.5%	0.06	1.2%	0.38	9.5%	6.60	5.7%	2.37	110.8%	0.83
CKG	5.68	3.5%	0.08	1.4%	0.06	1.5%	0.34	11.3%	5.64	6.9%	5.73	127.3%	0.60
CTU	5.85	6.2%	0.09	0.1%	0.06	0.1%	0.37	19.2%	5.96	11.1%	5.24	130.0%	0.73
DAC	5.91	3.1%	0.08	2.9%	0.06	1.4%	0.34	5.0%	5.92	5.8%	1.62	1.2%	0.73
DAR	7.46	47.2%	0.12	63.5%	0.09	69.7%	0.63	119.7%	9.04	85.6%	1.91	1729.5%	1.01
DEL	8.29	7.5%	0.13	3.8%	0.09	3.0%	0.74	14.0%	10.82	10.5%	4.23	110.5%	1.00
FIH	9.08	24.5%	0.14	31.8%	0.10	36.1%	0.87	56.2%	12.23	39.2%	3.14	1127.8%	1.17
HYD	8.82	14.9%	0.13	1.0%	0.09	0.9%	0.81	34.0%	11.61	21.3%	4.88	118.3%	1.21
IST	8.93	13.0%	0.14	9.2%	0.10	9.8%	0.89	25.9%	12.23	19.0%	11.64	17.5%	1.02
JKT	7.45	8.4%	0.11	8.0%	0.07	3.2%	0.51	9.9%	8.49	14.4%	3.55	97.2%	1.02
KHI	9.21	32.5%	0.15	32.4%	0.11	36.4%	0.95	65.5%	12.70	48.1%	6.71	143.8%	1.11
LAD	9.36	39.4%	0.15	48.5%	0.12	50.0%	1.06	64.3%	13.25	56.3%	4.78	145.8%	0.92
LAX	6.72	3.0%	0.10	4.4%	0.07	2.5%	0.45	4.2%	7.32	4.7%	14.31	39.5%	0.82
LHE	7.75	11.8%	0.12	11.7%	0.08	8.0%	0.63	16.2%	9.69	16.8%	3.73	72.3%	0.90
LIM	8.06	17.6%	0.14	12.2%	0.10	13.3%	0.83	39.4%	10.40	29.9%	5.99	38.1%	1.21
LON	8.30	6.1%	0.12	0.7%	0.08	0.5%	0.68	11.6%	10.47	8.5%	12.01	-17.4%	0.99
LOS	7.27	31.1%	0.11	38.9%	0.08	35.1%	0.55	58.5%	8.67	50.6%	2.50	1968.0%	0.83
MAA	7.02	2.9%	0.10	1.3%	0.07	0.8%	0.44	6.1%	7.98	4.2%	3.58	114.7%	0.96
MEX	9.54	19.1%	0.16	10.5%	0.12	11.1%	1.04	34.0%	13.64	26.2%	10.08	100.2%	1.19
MNL	7.68	16.9%	0.12	18.8%	0.09	19.3%	0.65	36.6%	9.72	27.3%	3.24	39.2%	0.90
MOW	7.98	5.9%	0.12	0.2%	0.08	0.1%	0.63	11.9%	9.88	8.3%	23.19	-28.9%	1.29
NKG	7.56	26.8%	0.11	0.0%	0.08	0.0%	0.70	55.0%	9.31	40.0%	9.28	126.8%	0.75
NYC	5.08	6.1%	0.07	6.8%	0.06	4.0%	0.27	14.9%	3.90	17.4%	14.64	36.6%	1.27
OSA	8.58	0.1%	0.14	0.1%	0.10	0.2%	0.80	0.4%	11.51	0.1%	14.80	-37.5%	0.86
PAR	8.32	5.0%	0.13	2.9%	0.09	2.5%	0.76	9.2%	10.75	7.3%	15.15	-6.6%	1.15
PEK	7.14	3.3%	0.11	0.2%	0.08	0.1%	0.53	6.5%	8.52	4.9%	5.94	122.5%	0.93
RIO	7.53	7.7%	0.12	6.3%	0.09	6.1%	0.63	16.1%	9.33	13.3%	6.36	42.5%	1.01
SAO	8.85	13.2%	0.14	7.5%	0.10	8.5%	0.81	26.2%	11.74	19.5%	8.95	41.8%	1.05
SEL	8.31	2.3%	0.13	1.3%	0.09	1.1%	0.73	3.7%	10.80	3.1%	25.98	-33.1%	0.80
SGN	6.52	13.8%	0.10	12.6%	0.08	8.9%	0.55	20.5%	7.72	21.8%	3.43	133.9%	0.93
SHA	7.17	4.6%	0.11	0.2%	0.08	0.1%	0.53	10.5%	8.58	6.9%	6.08	123.9%	0.72
SZX	6.46	5.6%	0.10	1.3%	0.07	1.2%	0.42	14.6%	6.86	9.8%	12.93	-25.4%	0.66
THR	8.55	10.9%	0.14	7.7%	0.10	6.8%	0.89	18.0%	11.64	16.1%	22.45	28.6%	0.99
TSN	7.30	4.3%	0.11	0.2%	0.08	0.2%	0.61	8.6%	8.99	6.1%	7.73	122.5%	0.90
TYO	9.04	0.1%	0.15	0.1%	0.10	0.1%	0.87	0.2%	12.25	0.1%	16.15	-37.4%	0.92

A.2 Empirical formulas for urban morphological parameters

We recite the formulas (Kanda et al., 2013; Varquez et al., 2017) used for calculating gridded urban morphological parameters in this study.

A.2.1 Average building height H_{avg}

For each grid, let P be the population density of that grid and $P_{\text{city max}}$ is the maximum population density of a grid in that city, define relative population density $RPD = P/P_{\text{city max}}$. Let $GDP_{city} = GDP_{country} \times (P_{city}/P_{country})$, H_{avg} is then estimated as

$$\alpha_{H_{avg}} = 2 \times 10^{-11} \times GDP_{city} + 9.80 \tag{A.1a}$$

$$\beta_{H_{avg}} = -2 \times 10^{-12} \times GDP_{city} + 9.18 \tag{A.1b}$$

$$H_{avg} = \alpha_{H_{avg}} \times RPD + \beta_{H_{avg}} \tag{A.1c}$$

A.2.2 Plan area index λ_p

$$\lambda_p = -0.2532 \times RPD^2 + 0.4562 \times RPD + 0.1125 \qquad (RPD \leq 0.9) \tag{A.2}$$

in which $(RPD \leq 0.9)$ means that if $RPD > 0.9$ then the value of 0.9 is used. The same notation is used in subsequent formulas.

A.2.3 Frontal area index λ_f

$$\lambda_f = 1.42\lambda_p^2 + 0.4\lambda_p \qquad (0.05 < \lambda_p < 0.45) \tag{A.3}$$

A.2.4 Zero-plane displacement height d

$$\sigma_H = 1.05H_{avg} - 3.7 \tag{A.4a}$$

$$H_{max} = 12.51\sigma_H^{0.77} \tag{A.4b}$$

$$X = \frac{\sigma_H + H_{avg}}{H_{max}} \qquad (0 \leq X \leq 1) \tag{A.4c}$$

$$\frac{d}{H_{max}} = -0.17X^2 + \left(1.29\lambda_p^{0.36} + 0.17\right) X \tag{A.4d}$$

where σ_H and H_{max} are standard deviation of building height and maximum building height, respectively.

A.2.5 Roughness length z_0

$$Y = \frac{\lambda_p \sigma_H}{H_{avg}} \qquad (Y \geq 0) \tag{A.5a}$$

$$\frac{z_0}{z_{0,mac}} = 20.21Y^2 - 0.77Y + 0.71 \tag{A.5b}$$

where $z_{0,mac}$ is estimated roughness length obtained from Macdonald equations (Macdonald et al., 1998):

$$\frac{d_{mac}}{H_{avg}} = 1 + A^{-\lambda_p}(\lambda_p - 1) \tag{A.6a}$$

$$\frac{z_{0,mac}}{H_{avg}} = \left(1 - \frac{d_{mac}}{H_{avg}}\right) \exp\left(-\left(0.5\beta\frac{C_D}{\kappa^2}\left(1 - \frac{d_{mac}}{H_{avg}}\right)\lambda_f\right)^{-0.5}\right) \tag{A.6b}$$

in which $A = 4.43$, $\beta = 1.0$, $C_D = 1.2$ is the nominal drag coefficient of a cubical obstacle, and $\kappa = 0.4$ is the von Kármán constant.

A.3 Domain configuration

Simulation was conducted with a fine domain (2 km resolution) nested in a coarse domain (10 km resolution). Data analysis (except for model verification) was performed for only a part of the fine domain because the minimum recommended domain size of 100 grids by 100 grids is much larger than the actual size of many cities. Analysis domains were chosen to capture metropolitan areas rather than the administrative borders of cities.

The domain configuration for each cities are drawn in the following pages. Cities are listed in lexicographical order. The maps were downloaded from Google Maps.

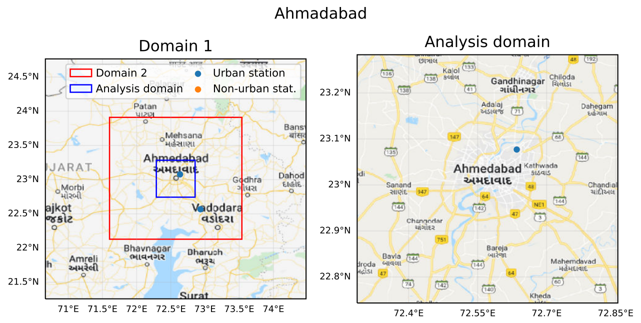

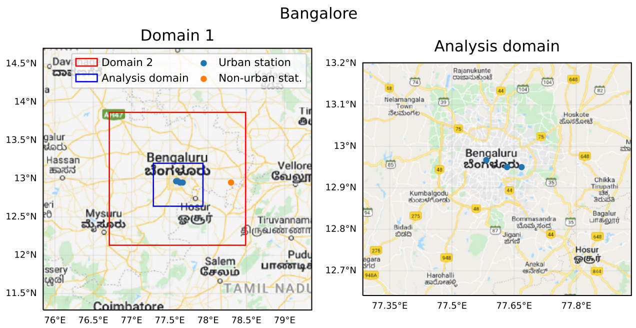

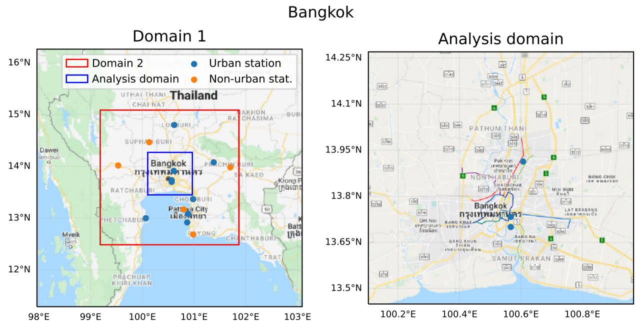

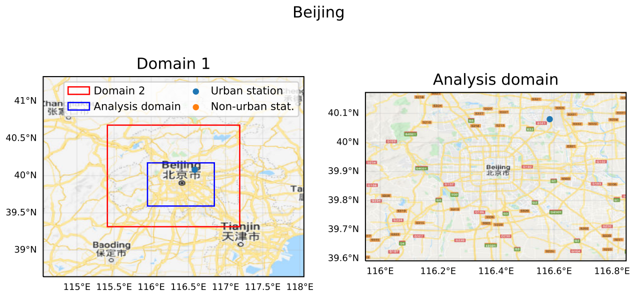

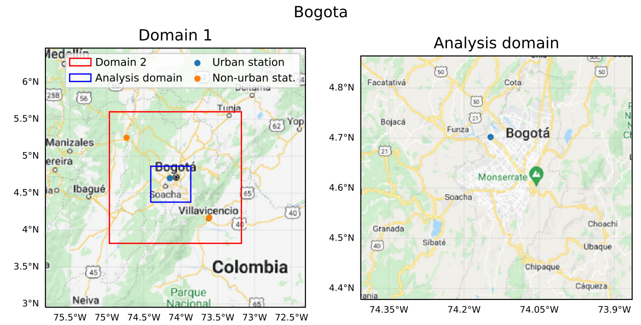

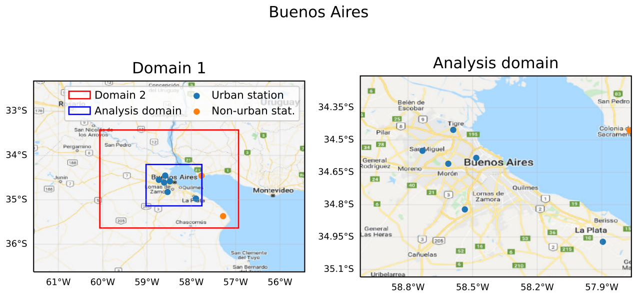

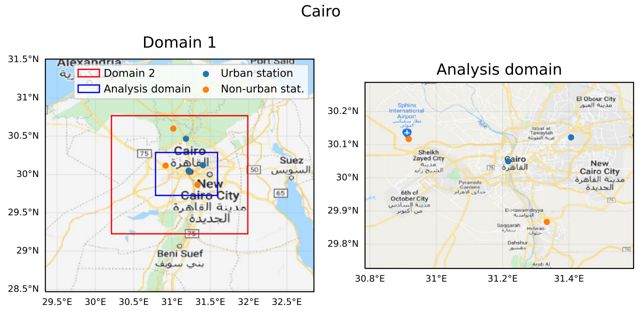

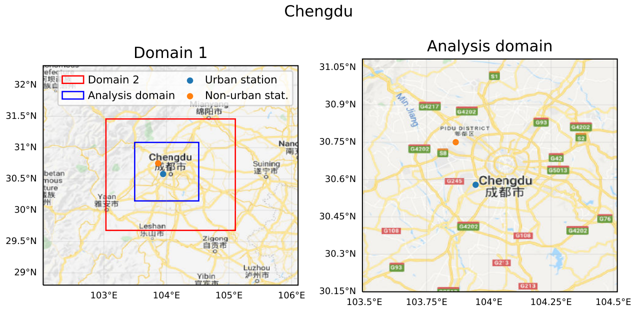

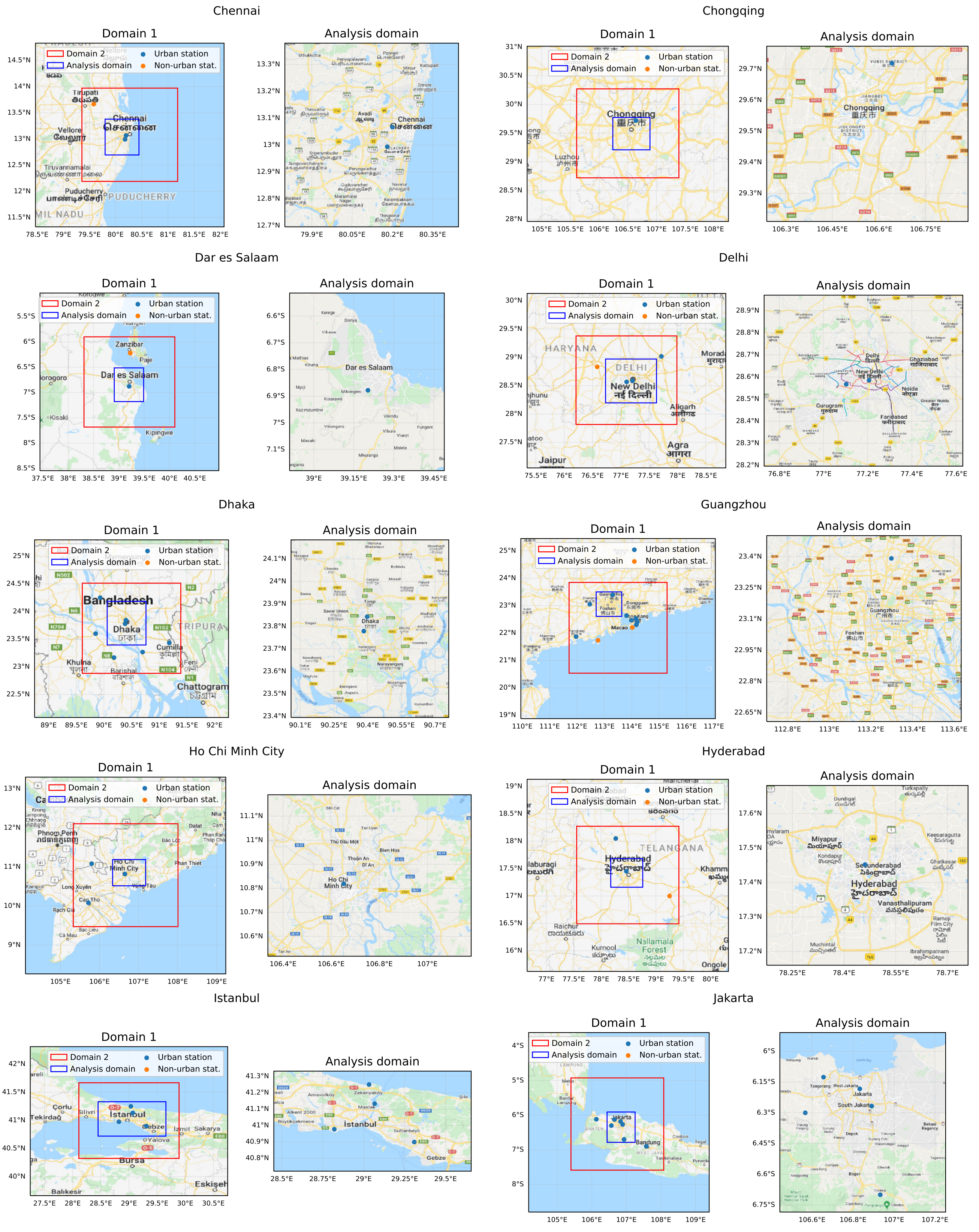

Chennai
Domain 1
Analysis domain
Domain 2
Analysis domain
Urban station
Non-urban stat.
Chongqing
Domain 1
Analysis domain
Dar es Salaam
Domain 1
Analysis domain
Delhi
Domain 1
Analysis domain
Dhaka
Domain 1
Analysis domain
Guangzhou
Domain 1
Analysis domain
Ho Chi Minh City
Domain 1
Analysis domain
Hyderabad
Domain 1
Analysis domain
Istanbul
Domain 1
Analysis domain
Jakarta
Domain 1
Analysis domain

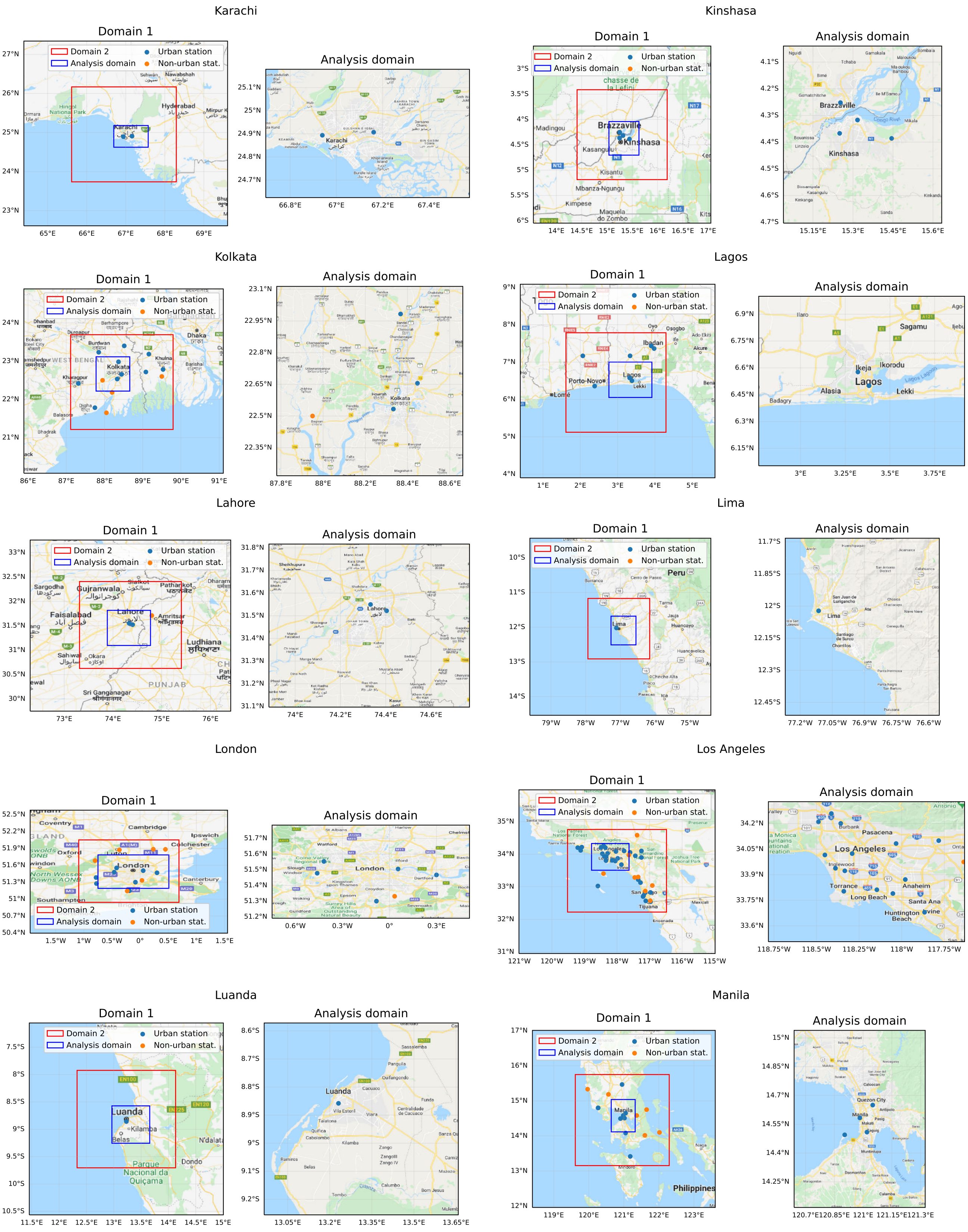
Karachi
Domain 1
Analysis domain
Kinshasa
Domain 1
Analysis domain
Kolkata
Domain 1
Analysis domain
Lagos
Domain 1
Analysis domain
Lahore
Domain 1
Analysis domain
Lima
Domain 1
Analysis domain
London
Domain 1
Analysis domain
Los Angeles
Domain 1
Analysis domain
Luanda
Domain 1
Analysis domain
Manila
Domain 1
Analysis domain
Domain 2
Analysis domain
Urban station
Non-urban stat.

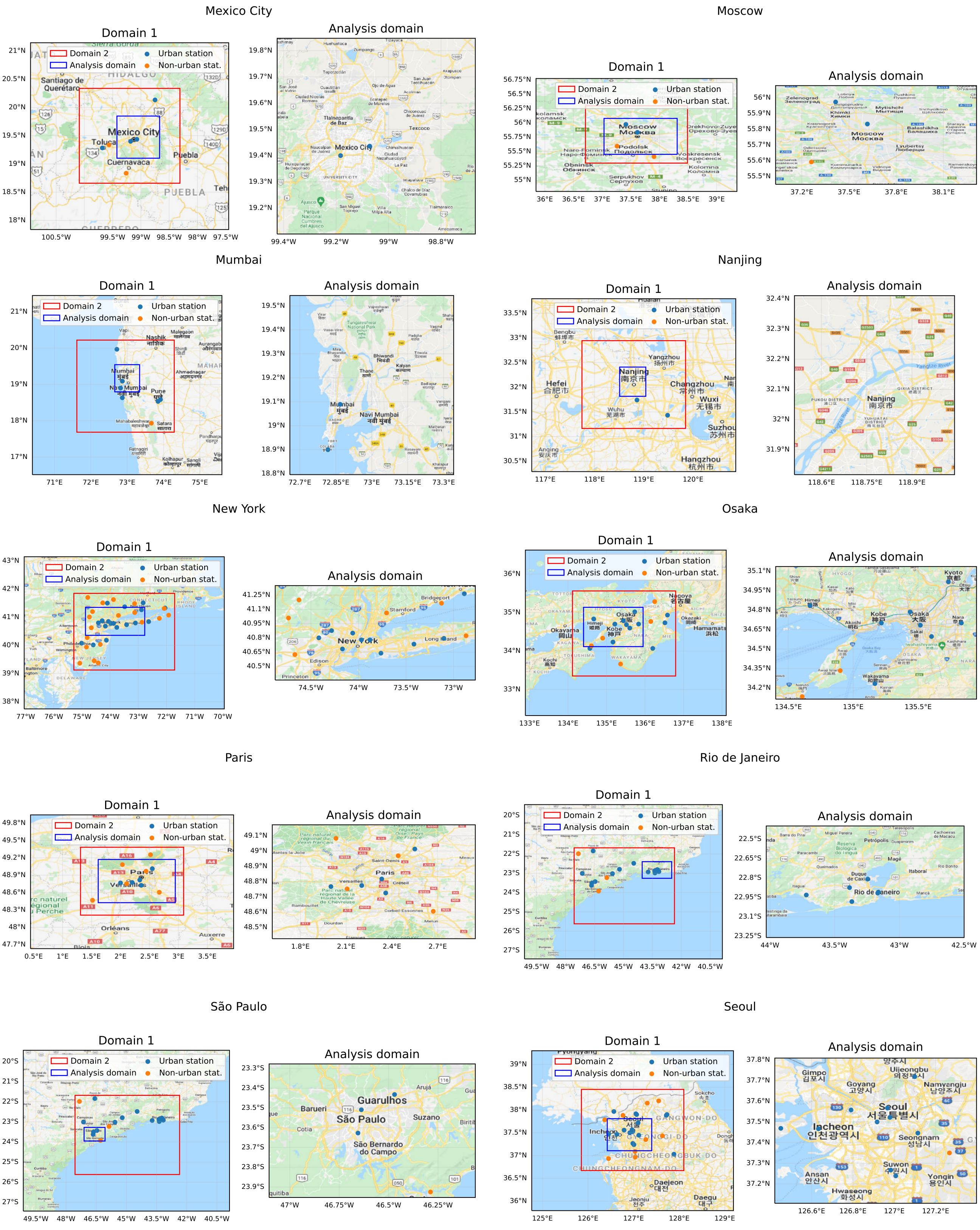
Mexico City
Domain 1
Domain 2
Analysis domain
Urban station
Non-urban stat.
Analysis domain
Moscow
Domain 1
Domain 2
Analysis domain
Urban station
Non-urban stat.
Analysis domain
Mumbai
Domain 1
Domain 2
Analysis domain
Urban station
Non-urban stat.
Analysis domain
Nanjing
Domain 1
Domain 2
Analysis domain
Urban station
Non-urban stat.
Analysis domain
New York
Domain 1
Domain 2
Analysis domain
Urban station
Non-urban stat.
Analysis domain
Osaka
Domain 1
Domain 2
Analysis domain
Urban station
Non-urban stat.
Analysis domain
Paris
Domain 1
Domain 2
Analysis domain
Urban station
Non-urban stat.
Analysis domain
Rio de Janeiro
Domain 1
Domain 2
Analysis domain
Urban station
Non-urban stat.
Analysis domain
São Paulo
Domain 1
Domain 2
Analysis domain
Urban station
Non-urban stat.
Analysis domain
Seoul
Domain 1
Domain 2
Analysis domain
Urban station
Non-urban stat.
Analysis domain

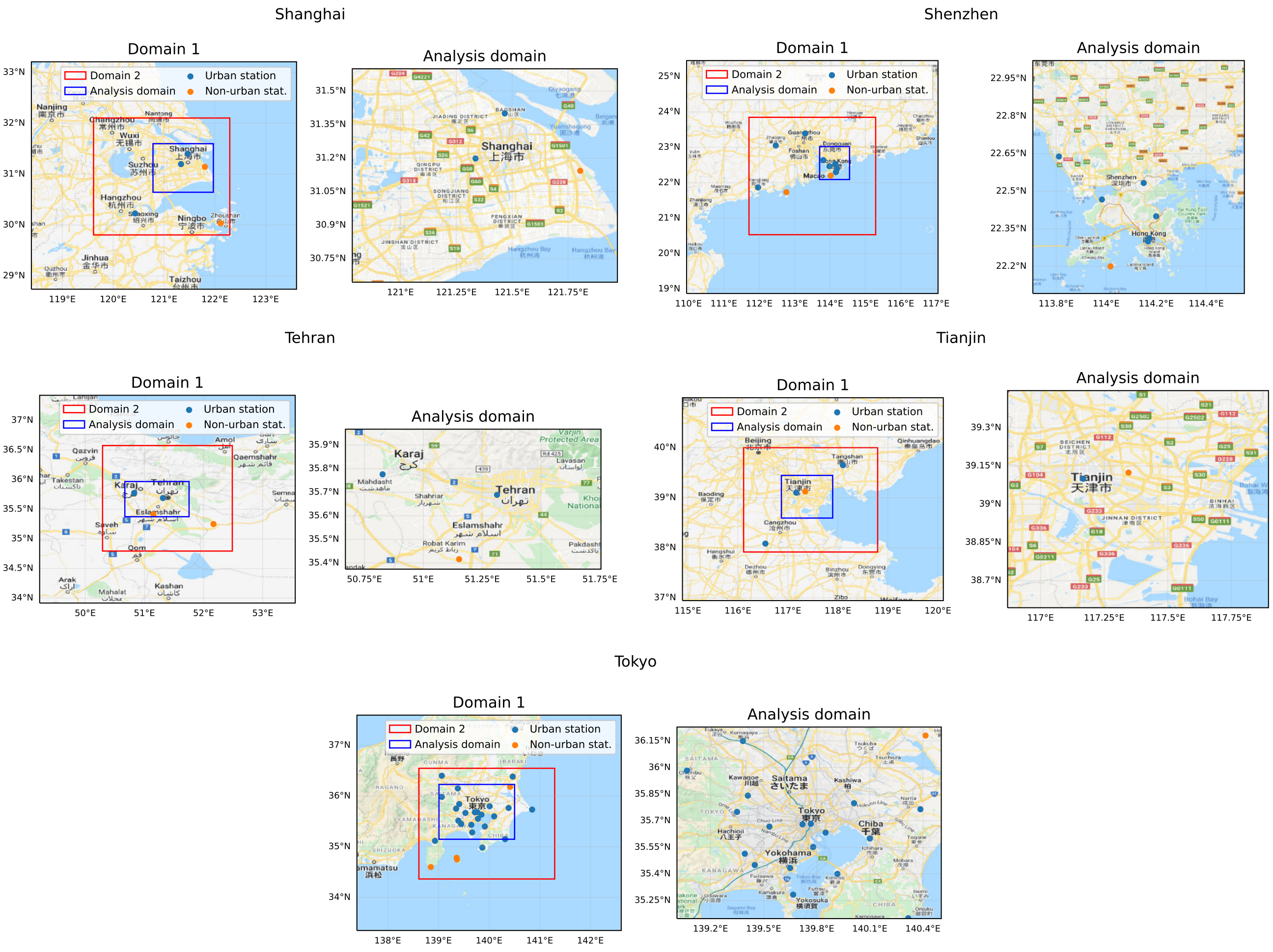

References

Kanda, M., Inagaki, A., Miyamoto, T., Gryschka, M., & Raasch, S. (2013). A new aerodynamic parametrization for real urban surfaces. *Bound.-Layer Meteorol.*, *148*(2), 357–377. https://doi.org/10.1007/s10546-013-9818-x

Macdonald, R. W., Griffiths, R. F., & Hall, D. J. (1998). An improved method for the estimation of surface roughness of obstacle arrays. *Atmospheric Environ.*, *32*(11), 1857–1864. https://doi.org/10.1016/s1352-2310(97)00403-2

Varquez, A. C. G., Darmanto, N., Kawano, N., Takakuwa, S., Kanda, M., & Xin, Z. (2017). Representative urban growing scenarios for future climate models. *J. Jpn. Soc. Civ. Eng., Ser. B1 (Hydraul. Eng.)*, *73*(4), I_103–I_108. https://doi.org/10.2208/jscejhe.73.i_103
